# Tumour-absorbed dose and efficacy of peptide receptor radionuclide therapy with [^90^Y]Y-DOTATOC in patients with refractory meningioma: a single-centre experience

**DOI:** 10.1007/s00259-026-08113-9

**Published:** 2026-08-05

**Authors:** Lilit Schweiger, Giulia Santo, Ariane Kronthaler, Gianpaolo di Santo, Sarah Iglseder, Stephanie Mangesius, Julian Mangesius, Katharina Stock, Irene Virgolini

**Affiliations:** 1 Department of Nuclear Medicine, Medical University of Innsbruck, Anichstr. 35, Innsbruck, 6020 Austria; 2 Department of Neurology, Medical University of Innsbruck, Innsbruck, Austria; 3 Department of Radiology, Medical University of Innsbruck, Innsbruck, Austria; 4 Neuroimaging Core Facility, Medical University of Innsbruck, Innsbruck, Austria; 5 Department of Radiation Oncology, Medical University of Innsbruck, Innsbruck, Austria

**Keywords:** Peptide receptor radionuclide therapy (PRRT), Tumour-absorbed dose, [^90^Y]Y-DOTATOC, Dosimetry

## Abstract

**Aim:**

The management of treatment-refractory meningioma in patients with previous surgical resection and radiation therapy remains challenging due to the lack of effective systemic treatment options. Therefore, novel therapeutic applications, such as peptide receptor radionuclide therapy (PRRT) targeting somatostatin receptors, may offer a promising therapeutic strategy. We aimed to assess the lesion-based tumour-absorbed dose and efficacy of PRRT with [^90^Y]Y-DOTATOC in patients with treatment-refractory meningioma.

**Materials and methods:**

In this study, 10 patients with therapy-refractory meningioma were retrospectively included. All patients received systemic [^90^Y]Y-DOTATOC therapy following prior surgical resection and radiation therapy. In total, 21 lesions were assessed morphologically by contrast-enhanced MRI and [^68^Ga]Ga-DOTATOC PET/CT both before and after therapy. All patients underwent dosimetry with [^111^In]In-DOTATOC prior to [^90^Y]Y-DOTATOC therapy. Treatment response was evaluated according to RANO bidimensional and volumetric criteria. Overall survival (OS) was defined from the first PRRT cycle until death.

**Results:**

The median cumulative activity administered per patient was 3804 MBq (IQR, 3140–4050 MBq), with a median of 3 cycles (range: 1–7). On lesion-based analysis following PRRT, response assessment was available in 19/21 (91.5%) lesions. Among these, tumour stabilisation according to RANO criteria was observed in 7/19 (36.8%) lesions, with a median cumulative tumour-absorbed dose of 52.7 Gy. Morphological tumour progression was noted in 12/19 (63.2%) lesions, with a median cumulative tumour-absorbed dose of 43.8 Gy. The difference in tumour-absorbed doses between morphologically stable and progressive lesions according to RANO criteria was not statistically significant (*P* = 0.82). Values for SUVmax and SUVmean from pretherapeutic [^68^Ga]Ga-DOTATOC PET/CT significantly correlated with the tumour-absorbed doses (*P* = 0.018 for SUVmax, *P* = 0.021 for SUVmean, respectively). Post-therapeutic [^68^Ga]Ga-DOTATOC PET/CT demonstrated an increase in SUVmax in 19/21 (90.5%) lesions (median: 40.8%, range: 13.0% − 101.2%). The median OS in the whole cohort was 23.0 months (95% CI 12.5–33.5 months).

**Conclusion:**

Our preliminary results indicate no significant difference in tumour-absorbed doses between morphologically stable lesions and those with progressive disease, as defined by the RANO criteria. However, lesion-based analysis revealed a significant correlation between pretherapeutic [^68^Ga]Ga-DOTATOC PET/CT uptake and tumour-absorbed doses following [^90^Y]Y-DOTATOC therapy. These findings suggest that baseline PET/CT may serve as a valuable tool for estimating cumulative tumour-absorbed dose and guiding the optimal cumulative activity of PRRT.

## Introduction

Meningiomas represent the most common primary brain tumours in adults, comprising roughly one-third of all primary central nervous system neoplasms [1]. These neoplasms are predominantly of benign origin and are preferably managed through complete surgical excision, often complemented by adjuvant radiotherapy when indicated. Nevertheless, in cases of therapy-refractory or recurrent disease — particularly in higher-grade meningiomas (WHO grade ≥ II) and tumours situated in anatomically critical locations — no established standard of care currently exists that demonstrates consistently favourable outcomes. Therefore, novel therapeutic approaches, including antihormonal therapy, cytotoxic agents, and peptide receptor radionuclide therapy (PRRT) targeting somatostatin receptors, have gained attention as emerging treatment modalities [2].

The molecular landscape of meningioma is defined by the overexpression of SSTR2a, which is observed in approximately 90–100% of cases [3–5]. This receptor expression enables a theranostic approach utilising radiolabelled somatostatin analogues for both high-resolution diagnostic PET imaging and subsequent targeted therapy [4, 6]. This framework establishes a salvage strategy for patients with progressive disease, allowing those with PET-confirmed somatostatin receptor expression to receive targeted therapy after standard surgical and radiotherapeutic options have been exhausted [7, 8]. While PRRT has demonstrated efficacy in gastroenteropancreatic neuroendocrine tumours, its application in meningioma requires careful optimisation [9, 10].

The most widely used therapeutic radionuclides for radiolabelling somatostatin analogues are the β-emitters yttrium-90 (^90^Y) and lutetium-177 (^177^Lu), typically administered every 6–10 weeks. β-emitters are characterised by a low linear energy transfer and a relatively long tissue penetration range, allowing the delivery of a relatively homogeneous radiation dose throughout the tumour [11]. Owing to its longer particle range and higher energy, ^90^Y is generally preferred for large-volume disease, whereas ^177^Lu is more suitable for small-volume lesions [11, 12].

Additionally, dosimetric assessment differs between the two radioisotopes. Radionuclides that emit γ-rays, such as ^177^Lu, enable the acquisition of both planar and three-dimensional SPECT images, allowing the distribution of radioactivity to be monitored over time. In contrast, ^90^Y is a pure β-emitter and does not emit γ-radiation. Therefore, surrogate radionuclides, such as ^111^In, are often used for dosimetric evaluation [13].

PRRT is generally administered at a fixed activity per cycle, although this standardised approach may not ensure that each patient receives an effective absorbed dose to the tumour.

In the present study, individualised patient-specific dosimetry based on the Medical Internal Radiation Dose (MIRD) framework was performed to quantify the tumour absorbed dose delivered by standard treatment approach [14]. The aim was to investigate the correlation between lesion-based tumour doses with response and baseline imaging parameters in a single-centre cohort of patients with advanced/refractory meningioma treated with [^90^Y]Y-DOTATOC PRRT. Survival outcome and safety profile were also reported.

## Materials and methods

### Patient cohort

In total, ten patients (6 women, 4 men; mean age 58,7 ± 7,9y) with histologically confirmed and radiologically progressive refractory meningioma were included. All patients had progressed despite surgery and external beam radiotherapy (EBRT). Detailed patient characteristics are presented in Table 1. At the time of PRRT, all patients presented with recurrent meningioma. Histopathological evidence of brain invasion was present in two patients. Metastatic disease was observed in two patients, one of whom also had histopathologically confirmed brain invasion. Metastatic sites included the lungs, mediastinal lymph nodes and bone.

**Table 1:** Patient characteristics

Parameter	Respective Counts (*n*) and Percentages (%)
No. of patients	10
Mean Age (Years)	58,7 ± 7,9y
Sex (Female/Male)	6/4
Salvage radiotherapy after resection	10 (100%)
Histology	
CNS WHO Grade 1	0 (0%)
CNS WHO Grade 2	3 (30%)
CNS WHO Grade 3	6 (60%)
Unknown WHO Grading	1 (10%)
Lesion Localisation	
Skull Base/Sphenoid Wing	8 (38.1%)
Cavernous Sinus/Para-sellar	4 (19%)
Parafalcine/Parietal	3 (14.3%)
Other/Meningiomatosis	2 (9.6%)
Mean time between last PRRT cycle and follow-up imaging (months)	2.6 (range: 2.4–3.3)

Documented evidence of progressive disease (PD) according to RANO criteria was required for all patients prior to study enrolment to confirm the treatment-refractory status [9]. All patients demonstrated high SSTR2a expression on baseline [^68^Ga]Ga-DOTATOC PET/CT, with target lesion uptake significantly exceeding that of intracranial reference structures.

**Fig. 1:**
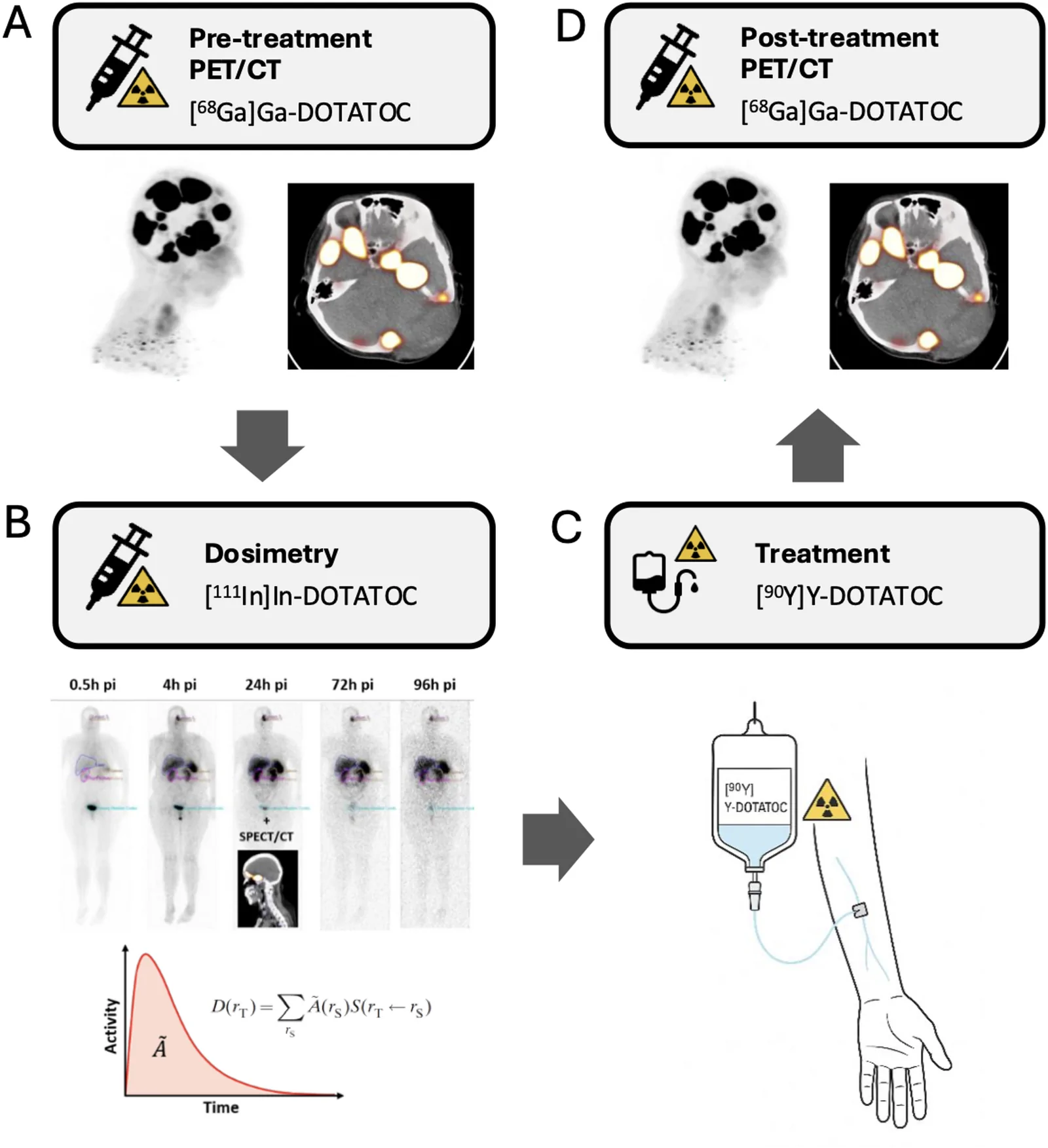
(**A**) Pre-treatment [^68^Ga]Ga-DOTATOC PET/CT showing multiple intracranial meningiomas. (**B**) Patient-specific dosimetry using [^111^In]In-DOTATOC planar imaging with dose estimation by MIRD schema. (**C**) Intravenous application of [^90^Y]Y-DOTATOC (~ 3804 MBq). (**D**) Post-treatment PET/CT demonstrating stable disease with persistent tracer uptake and no evidence of newly developed tumour lesions

The overall study workflow, including imaging, dosimetry, therapy, and follow-up assessment, is summarised in Fig. 1.

### PET/CT Imaging

Pre- and post-therapeutic PET/CT imaging of the brain covering the vertex to the skull base was performed on a dedicated time-of-flight system (Discovery MI; GE Healthcare). The PET scan was acquired in three-dimensional mode approximately 1 h after intravenous injection of 100 MBq of [^68^Ga]Ga-DOTATOC, using an axial field of view of 20 cm and an acquisition time of 2 min per bed position. A low-dose CT scan was obtained for attenuation correction of the PET emission data. PET images were corrected for random coincidences, scatter, and radioactive decay. Image reconstruction was performed using the VUE Point FX iterative reconstruction algorithm (GE Healthcare) without z-axis filtering, combined with the Q.Clear reconstruction algorithm (β = 1,000; GE Healthcare), a fully convergent Bayesian penalized likelihood reconstruction method with noise control.

### Dosimetry

Individualised dosimetry was performed using [^111^In]In-DOTATOC as a surrogate tracer for the biodistribution of [^90^Y]Y-DOTATOC. Following administration of [^111^In]In-DOTATOC, planar scintigraphy was acquired at approximately 30 min, 4 h, 24 h, 72 h, and 96 h post-injection. From these serial measurements, time–activity curves (TACs) were generated to determine the time-integrated activity (Ã) for the tumour volume and organs at risk (OARs), specifically the kidneys and bone marrow. Absorbed doses (D) were subsequently calculated according to the MIRD formalism (D = Ã * S), allowing personalised treatment with respect to organ dose constraints [15].

### Therapeutic intervention

[^90^Y]Y-DOTATOC was synthesised at the Department of Nuclear Medicine, Medical University of Innsbruck, using a fully automated radiolabelling system according to the institutional protocol established at our centre and based on the method described by Petrik et al. [16]. Routine quality control was performed before release for clinical administration.

Patients received PRRT with [^90^Y]Y-DOTATOC, which was selected based on the volume of the lesion(s) [11, 12].

Therapy cycles were administered every 10–12 weeks, with blood counts and renal function parameters monitored after one week and every 4 weeks. To prevent nephrotoxicity, a lysine/arginine amino acid solution was infused over 4 h simultaneously with the radioligand.

### Follow-up and volumetric assessment

MRI (1.5/3T) sequences included T1 (+/- contrast), T2, and FLAIR. 3D segmentation was performed using Brainlab Elements SmartBrush. Response was adjudicated via RANO-M criteria: Response assessment strictly adhered to the RANO meningioma framework, where PD was defined as a ≥ 40% increase in volume and partial response (PR) required a ≥ 65% decrease in volume [9].

### Statistical analysis

Statistical analyses were performed using IBM SPSS Statistics (version 31.0) and GraphPad Prism (version 11.0). Descriptive statistics for continuous variables are presented as median and range (or interquartile range) due to the small cohort size and non-normal distribution of the data. Differences in cumulative tumour-absorbed doses between morphological response groups (Stable Disease (SD) vs. PD) were analysed using the non-parametric Mann-Whitney U test. Correlations between pre-therapeutic imaging metrics (SUVmax, SUVmean, and volume) and the calculated tumour-absorbed doses were assessed using the Spearman rank correlation test. Overall survival (OS) was calculated from the initiation of PRRT to the date of death or the date of last follow-up for censored patients using the Kaplan–Meier method. For all tests, a P-value < 0.05 was considered to indicate statistical significance.

## Results

### Treatment administration and clinical outcomes

[^90^Y]Y-DOTATOC was administered with a median cumulative activity of 3804 MBq (IQR, 3140–4050 MBq). Patients received a median of 3 cycles (range: 1–7) of PRRT.

In total, 21 lesions were followed; however, radiological response assessment according to the RANO criteria was eligible in 19 out of 21 target lesions (91.5%); the remaining two lesions were not evaluable as they had undergone interim resection prior to follow-up imaging. Within this cohort, tumour stabilisation (SD) was observed in 7/19 lesions (36.8%). The remaining 12/19 lesions (63.2%) demonstrated morphological tumour progression according to RANO-criteria at response assessment after treatment.

### Tumour dosimetry and response assessment

**Fig. 2:**
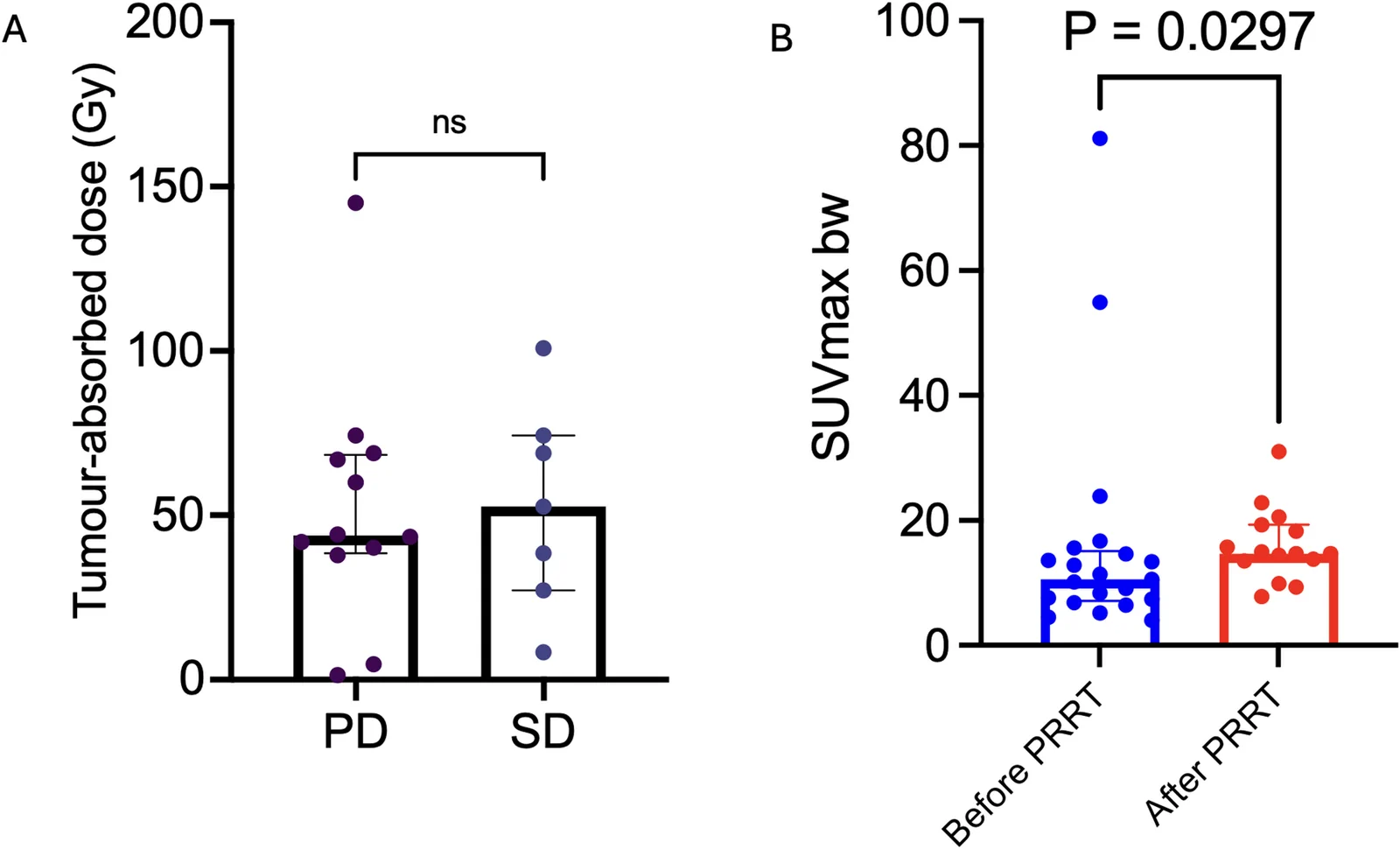
(**A**) Stable lesions showed higher absorbed doses without statistical significance (*P* = 0.82). (**B**) Lesion-based analysis revealed significantly increased [^68^Ga]Ga-DOTATOC uptake in nearly all lesions following [^90^Y]Y-DOTATOC PRRT

Patient-specific dosimetry using [^111^In]In-DOTATOC imaging allowed for the calculation of cumulative tumour-absorbed doses. Considering all lesions, the median cumulative absorbed dose was 48.5 Gy (IQR, 38.9–73.0 Gy), corresponding to a median absorbed dose coefficient of 4.7 Gy/GBq (IQR, 2.5–7.4 Gy/GBq). Lesions meeting the RANO criteria for SD received a median cumulative absorbed dose of 52.7 Gy (IQR 38.5–74.3 Gy). In contrast, lesions with morphological progression (PD) received a median cumulative absorbed dose of 43.8 Gy (IQR, 38.4–72.5 Gy). Although stable lesions received a higher median absorbed dose, the difference in tumour-absorbed doses between the stable and progressive groups was not statistically significant (*P* = 0.82) (Fig. 2A).

### SSTR PET: Baseline and follow-up analysis

**Fig. 3:**
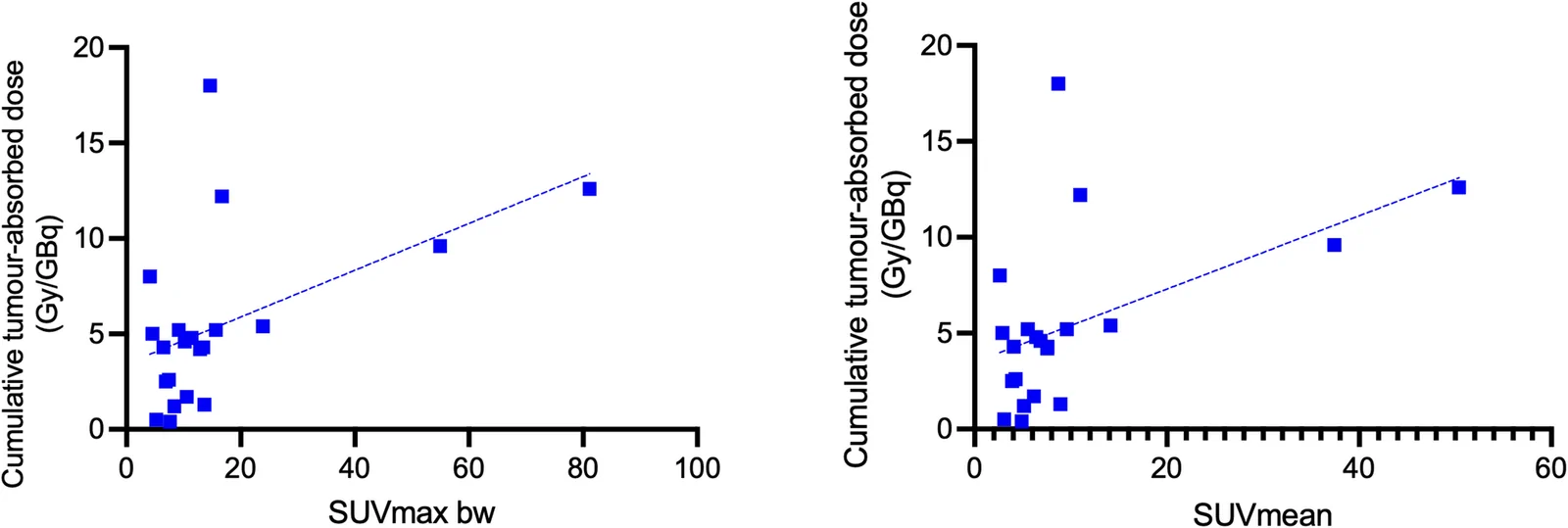
Pre-therapeutic [^68^Ga]Ga-DOTATOC PET/CT SUVmax and SUVmean correlated significantly with tumour-absorbed dose (*P* = 0.018 and *P* = 0.02, respectively)

Analysis of baseline somatostatin receptor expression via [^68^Ga]Ga-DOTATOC PET/CT revealed that semi-quantitative uptake values were strong predictors of the delivered dose. Both SUVmax and SUVmean from the pre-therapeutic scans showed significant positive correlations with the calculated tumour-absorbed doses (*P* = 0.018 for SUVmax, *P* = 0.021 for SUVmean, respectively) (Fig. 3). None of the volumetric parameters derived from pre-treatment [^68^Ga]Ga-DOTATOC PET or MRI were correlated with treatment response according to RANO (data not shown).

Moreover, post-therapeutic follow-up with [^68^Ga]Ga-DOTATOC PET/CT revealed a marked increase in SUVmax in 19 out of 21 lesions (90.5%) (Fig. 2B). The median increase in uptake was 40.8% (range: 13.0% – 101.2%) relative to pre-therapeutic levels. This significantly increased tracer uptake was observed across nearly all lesions following therapy.

**Fig. 4:**
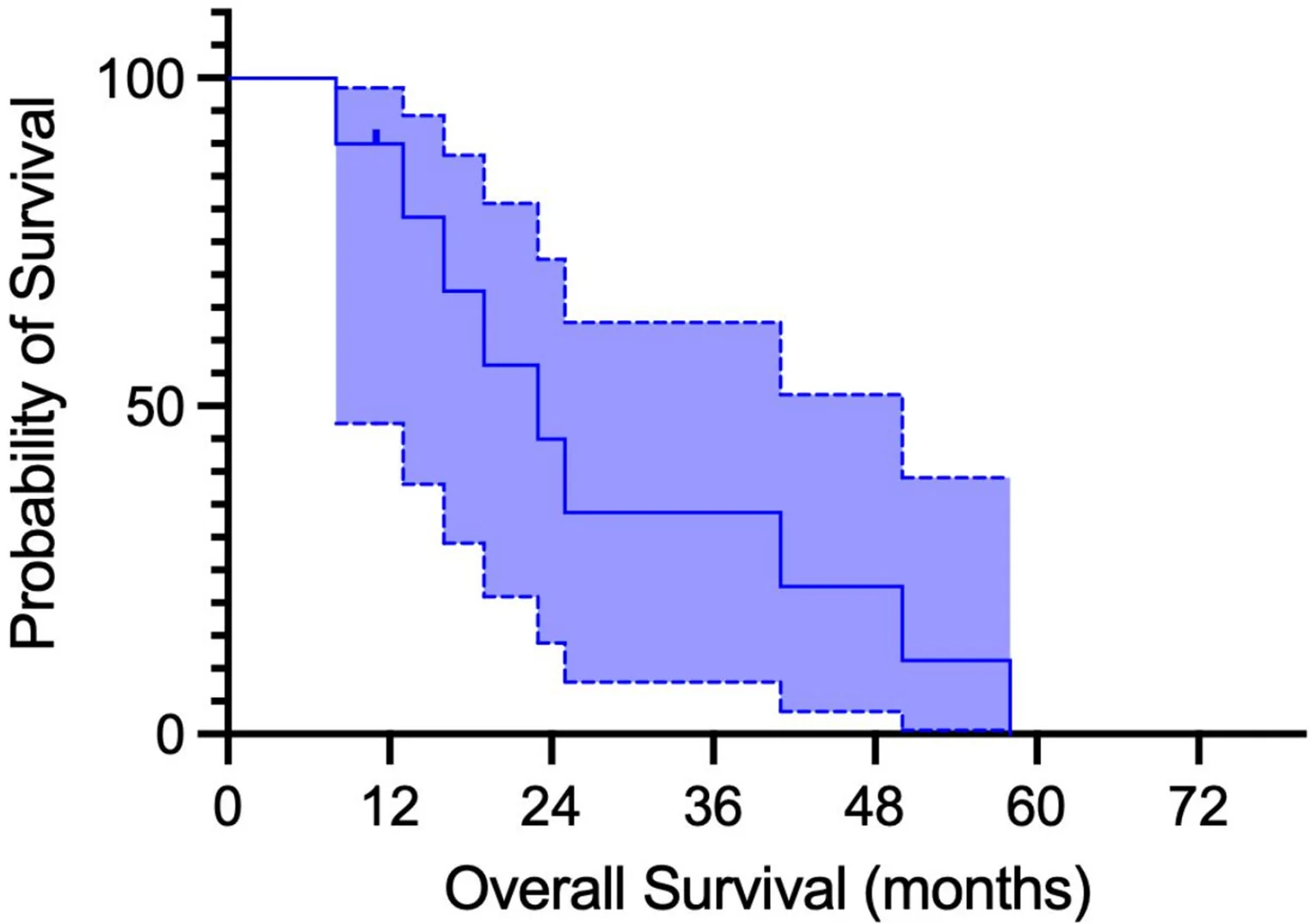
Median overall survival (Kaplan-Meier method) from the beginning of [^90^Y]Y-DOTATOC treatment to death or latest follow-up was 23 months

Clinical follow-up for the cohort demonstrated a median overall survival (OS) of 23.0 months. The 95% confidence interval for overall survival ranged from 12.5 to 33.5 months (Fig. 4).

**Fig. 5:**
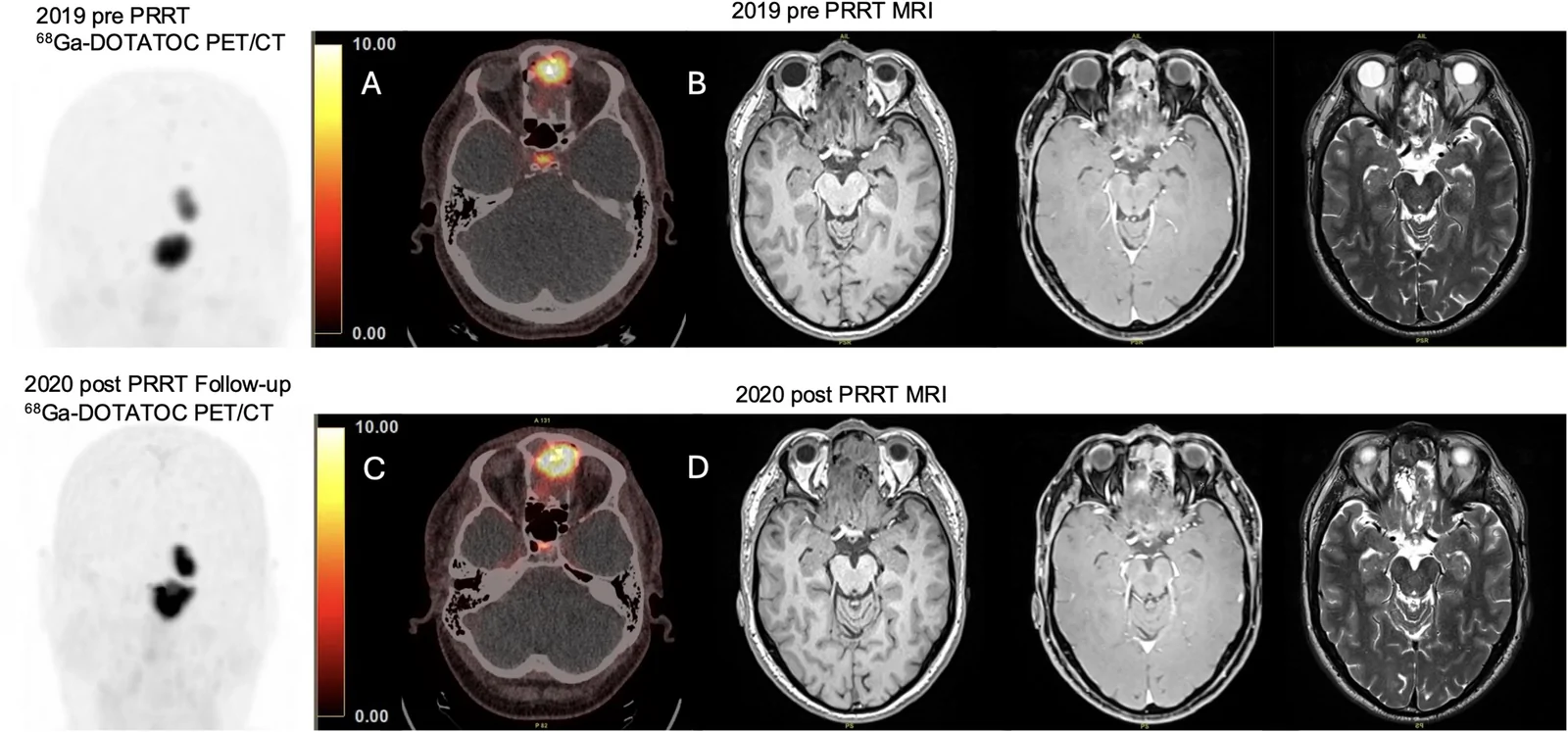
(**A** - **B**) [^68^Ga]Ga-DOTATOC PET/CT and MRI demonstrating recurrent anaplastic olfactory meningioma (WHO grade III) in a patient previously treated with stereotactic radiosurgery (2014), fractionated SECTOR radiotherapy and proton therapy (2015). (**C** - **D**) Follow-up [^68^Ga]Ga-DOTATOC PET/CT and MRI after enrolment in our [^90^Y]Y-DOTATOC protocol: after three cycles of [^90^Y]Y-DOTATOC PRRT, the patient achieved stable disease by RANO criteria with clinical improvement

One representative patient example is presented in Fig. 5.

### Toxicity profile

Treatment-related toxicity was retrospectively assessed according to the Common Terminology Criteria for Adverse Events (CTCAE), version 6.0, using the worst post-baseline laboratory values recorded during PRRT and within the available follow-up after the last PRRT cycle. Overall, PRRT with [^90^Y]Y-DOTATOC was well tolerated, and no CTCAE grade 4 or grade 5 adverse events occurred.

Haematological toxicity represented the most common adverse event. Anaemia was observed in five patients, consisting of grade 1 anaemia (*n* = 4) and grade 3 anaemia (*n* = 1). Leukopenia occurred in six patients, including grade 1 leukopenia (*n* = 3) and grade 2 leukopenia (*n* = 3). Neutropenia was documented in two patients, comprising grade 1 neutropenia (*n* = 1) and grade 2 neutropenia (*n* = 1). Thrombocytopenia occurred in three patients, including grade 1 thrombocytopenia (*n* = 2) and grade 2 thrombocytopenia (*n* = 1).

Renal toxicity was limited to grade 1 creatinine elevation (*n* = 3), and no clinically relevant nephrotoxicity was observed. Mild hepatic toxicity was observed, consisting exclusively of grade 1 elevations of AST (*n* = 2), ALT (*n* = 2), and total bilirubin (*n* = 1).

Clinically, treatment was generally well tolerated. One patient experienced transient nausea and vomiting associated with orthostatic collapse, which resolved completely with conservative management. No patient required treatment discontinuation or dose reduction because of treatment-related toxicity.

Across all 10 patients, the median cumulative kidney absorbed dose over all PRRT cycles was 19.3 Gy (IQR, 16.1–22.5 Gy). The median cumulative bone marrow absorbed dose was 0.8 Gy (IQR, 0.5–1.9 Gy).

## Discussion

The management of treatment-refractory meningioma remains a significant clinical challenge due to the lack of established systemic therapies once local options are exhausted. In this study, we evaluated the therapeutic efficacy and dosimetric characteristics of [^90^Y]Y-DOTATOC PRRT in patients with therapy-refractory meningioma.

The observed SD rate of 36.8% and PD rate of 63.2% underscore the profound biological resistance inherent in advanced/refractory meningioma. Our findings align with the data reported by Minczeles et al. [3], who noted a disease control rate of approximately 40%. In contrast, Bartolomei et al., in 29 meningioma patients who underwent [^90^Y]Y-DOTATOC, showed disease stabilisation in 19 of 29 patients (66%) and progressive disease in the remaining 10 (34%). In that study, outcomes were significantly better in patients with WHO grade I meningiomas than in those with grade II–III disease, with a median time to progression of 61 months and 13 months, respectively [5].

The higher PD rate observed in our cohort compared with previous studies evaluating either [^90^Y]Y-DOTATOC alone [17] or tandem ^90^Y/^177^Lu PRRT [18] is likely explained by the particularly advanced and heavily pre-treated nature of our patient population. Repeated failures of surgical resection and EBRT may have selected for biologically more aggressive tumour clones with reduced susceptibility to PRRT. Furthermore, our cohort was enriched for high-grade disease, with WHO grade III meningiomas accounting for approximately 60% of cases, which likely contributed to the poorer treatment outcomes.

Despite the lack of objective tumour responses, the median OS of 23.0 months is clinically meaningful in the context of salvage setting, particularly considering the patients’ baseline characteristics. This survival duration is consistent with the long-term follow-up analysis by Severi et al. [18], who reported a median progression-free survival (PFS) of 16 months and median OS of 36 months in 42 patients treated with [^90^Y]Y-DOTATOC or [^177^Lu]Lu-DOTATATE. Similar survival outcomes have also been reported in other cohorts homogeneously treated with [^90^Y]Y-DOTATOC [17], further supporting PRRT as a viable therapeutic option for achieving disease stabilisation in patients who have exhausted standard treatment options.

The absence of a statistically significant difference in absorbed dose between SD and PD lesions (*P* = 0.82) suggests that in refractory cases, the total absorbed dose delivered (e.g., 52.7 Gy) may be secondary to tumour-intrinsic factors. As Boursier et al. [19] proposed, SSTR heterogeneity and the stochastic effects of radiation in small lesions may limit the efficacy of ^90^Y, despite its long path length.

To the best of our knowledge, no comparable dosimetric studies reporting tumour absorbed doses following [^90^Y]Y-DOTATOC therapy in meningioma are currently available. Therefore, comparison is limited to the available literature on [^177^Lu]Lu-labelled PRRT, in which the median cumulative tumour absorbed dose observed in our cohort (48.5 Gy) appears higher than those generally reported for conventional intravenous treatment with [^177^Lu]Lu-DOTATATE or [^177^Lu]Lu-DOTATOC. A recent review summarised cumulative tumour absorbed doses of approximately 1–10 Gy for standard fixed-activity regimens [20]. Only one of the few available dosimetric studies reported a higher median cumulative tumour absorbed dose of 36 Gy, although these estimates were obtained using a simplified dosimetric methodology [3]. However, direct comparison between studies is limited by substantial differences in radionuclide, treatment regimen, and dosimetric methodology.

Another important consideration is that the present dosimetry was based on pre-therapeutic [¹¹¹In]In-DOTATOC imaging. Although it was suggested that dosimetry based on ^111^In-labelled compounds does not reliably correlate with the biological effects of ^90^Y-based treatments [21], it represents a simple and reproducible clinical surrogate for pre-therapeutic dosimetric calculations.

Of note, most published studies have focused on ^177^Lu-labelled SSTR ligands, particularly [^177^Lu]Lu-DOTATATE, whereas ^90^Y-based PRRT have been used only in a minority of studies. Nevertheless, a recent meta-analysis [22] reported that, in subgroup analyses stratified by radiopharmaceutical type, no significant differences were observed between ^177^Lu- and ^90^Y-labelled SSTR ligands in terms of disease control rate.

The correlation between pre-therapeutic SUVmax/SUVmean and tumour-absorbed dose (*P* = 0.018 for SUVmax, *P* = 0.021 for SUVmean, respectively) underlines the predictive value of SSTR-PET/CT in treatment planning. This supports the clinical frameworks established by Albert et al. [23] and Hänscheid et al. [24], demonstrating that molecular imaging effectively predicts SSTR-receptor binding and subsequent cellular internalisation, which are required for effective dose deposition.

Furthermore, our findings confirm that baseline PET metrics can guide individualised treatment planning, allowing for the optimisation of cumulative activity based on predicted lesional uptake.

In addition, a median increase of 40.8% in SUVmax was observed after [^90^Y]Y-DOTATOC PRRT across almost all lesions. In a prospective phase II multicentre study including 14 patients with progressive meningioma, Kurz et al. suggested that [^68^Ga]Ga-DOTATATE uptake may serve as an imaging biomarker for treatment response assessment [25]. These findings support the hypothesis that PET imaging could potentially provide early indications of treatment response or disease progression, also for lesions that appear stable on MRI. Namely, while recent evidence suggests that higher pre-treatment SSTR2 expression, assessed by both SSTR-PET and immunohistochemistry, is associated with improved treatment response to PRRT [26], there is currently no evidence that increased post-treatment SSTR-PET uptake represents a possible treatment-related biological effect in recurrent meningioma.

The favourable safety profile observed in our cohort is in line with the published experience of intravenous [^90^Y]Y-DOTATOC PRRT in recurrent meningioma. In the present study, treatment-related toxicity was predominantly mild, with haematological adverse events representing the most frequent laboratory abnormalities. Importantly, no CTCAE grade 4 or 5 toxicities occurred, no clinically relevant nephrotoxicity was observed, and no patient required treatment discontinuation or dose reduction because of treatment-related adverse events.

Similar findings were reported by Bartolomei et al. [5], who described good overall treatment tolerability, predominantly reversible grade 1 white blood cell toxicity affecting 18 of 29 patients, and only a single case of permanent grade 1 renal toxicity despite repeated treatment cycles.

Likewise, Gerster-Gilliéron et al. [17] reported that intravenous administration of [^90^Y]Y-DOTATOC was generally well tolerated, with predominantly transient haematological toxicity, only one patient developing transient grade 2 renal toxicity, and mild transient elevations of hepatic enzymes without a clear causal relationship to treatment.

Taken together, these findings consistently support the favourable safety profile of intravenous [^90^Y]Y-DOTATOC, even in heavily pre-treated patients with limited therapeutic alternatives.

Consistent with these observations, Zhang et al. [27] recently reported long-term outcomes following PRRT in patients with refractory meningioma, demonstrating disease control in 85.7% of evaluable patients, a median progression-free survival of 32.3 months, and an excellent long-term safety profile without CTCAE grade 2–4 haematological toxicity, hepatotoxicity, or late radiation-related adverse effects.

Interestingly, while our IV-administration yielded moderate stabilisation, it contrasts sharply with emerging intraarterial (IA) results. Amerein et al. (2024) [28] reported a superior 6-month PFS of 76.9% and a 15.4% objective remission rate (1 CR, 1 PR in patients) using IA [^177^Lu]Lu-HA-DOTATATE. The IA route likely bypasses first-pass metabolism and achieves higher receptor saturation. Future strategies should consider IA administration or the combination therapies proposed by Kreissl et al. [10] to overcome the biological hurdles of refractory WHO grade II/III lesions.

This study is limited by its small sample size (*N* = 10) and its focus on a highly heterogeneous, end-stage refractory population with disease progression according to RANO. These factors restrict the generalisability of the findings to treatment-naïve patients or those with slower-growing, low-grade (WHO Grade I) disease. Another limitation of this study is the lesion-based analysis without accounting for intra-patient correlation. Since multiple lesions from the same patient were treated as independent observations, variance may have been underestimated and statistical significance potentially overestimated.

## Conclusion

In conclusion, [^90^Y]Y-DOTATOC PRRT is a potential salvage therapy for treatment-refractory meningioma, providing a meaningful median OS of 23.0 months with about 40% of stable lesions in our heavily pretreated, high-grade cohort. However, no difference was shown in tumour dose between stable and progressive lesions. Crucially, the significant correlation between pre-therapeutic PET metrics and delivered radiation doses identifies baseline [^68^Ga]Ga-DOTATOC PET/CT as a reliable predictive biomarker. Individualised activity calculation may help to optimise dose delivery, potentially enabling higher tumour-absorbed doses and improving therapeutic efficacy in this challenging clinical setting.
